# Peripheral interstitial lung abnormalities on LDCT in an asymptomatic, nonsmoking Chinese urban cohort

**DOI:** 10.1097/MD.0000000000033630

**Published:** 2023-04-21

**Authors:** Zhimei Gao, Xin Li, Yan Li, Chenguang Zhang, Yaguang Li, Mengyue Sun, Yalan Wu, Shujing Li, Yingqi Zhang

**Affiliations:** a The Department of Radiology and Nuclear Medicine, The First Hospital of Hebei Medical University, Shijiazhuang, China; b The Department of CT, Tangshan Workers Hospital, Tangshan, China; c The Department of CT and MRI, The Children’s Hospital of Hebei Province, Shijiazhuang, China; d The Department of Emergency, The First Hospital of Hebei Medical University, Shijiazhuang, China

**Keywords:** aging, low-dose computed tomography, lung, peripheral interstitial lung abnormalities

## Abstract

To retrospectively investigate the imaging features and the related influencing factors of peripheral interstitial lung abnormalities (PILA) that caused “normal aging” by low-dose computed tomography (LDCT) in an nonsmoking, asymptomatic Chinese urban cohort. The clinical data of 733 subjects who underwent chest LDCT were retrospectively collected. The computed tomography (CT) signs of PILA (interlobular septal thickening [ILST], intralobular interstitial thickening [ILIT], ground-glass opacity [GGO], reticular shadow [RS], subpleural line [SL]) were evaluated at 6 levels and statistically analyzed. The effects of age, sex, body mass index (BMI), blood pressure (BP), and blood biochemistry parameters on ILST, ILIT, and RS were analyzed by Binary Logistic regression analysis. Significant age differences in PILA were found. None of the 5 PILA CT signs (GGO, ILST, ILIT, RS, and SL) was observed in subjects under 40 years old, while in subjects over 40 years old, the incidence of PILA increased with age. All 5 CT signs of PILA were significantly different among the subjects aged 18 to 49, 50 to 69, and 70 to 79 (*P* < .05). There was no significant sex difference in PILA. Among age, sex, BMI, BP, and laboratory biochemistry parameters, only age had a significant effect on ILST, ILIT, and RS. LDCT can be used as a noninvasive method to evaluate the PILA. PILA were mainly affected by age, while sex, BMI, BP, and laboratory biochemistry parameters had little effect on PILA. PILA observed before the age of 40 years should be considered an abnormal finding, whereas it is common in individuals over 70.

## 1. Introduction

In the past few decades, human life expectancy and the proportion of the elderly population (especially people over 65 years old) have greatly increased. However, the trend has not been accompanied by a similar increase in their healthspan.^[1,2]^ Healthspan is the period of our life without major debilitating diseases.^[3]^ Increasing healthspan can extend the healthy period of life and delay the development of chronic diseases and disability. Optimal longevity (living long, and with good health and quality of life) is considered as an important way to solve the problem of population aging.^[4]^ Therefore, it will become particularly important to distinguish “diseases” of each organ system including respiratory system for “healthy” aging.

The interstitial lung diseases (ILD) comprise a diverse group of lung diseases with overlapping clinical, radiological, physiological, and pathological features. Idiopathic pulmonary fibrosis (IPF) is one of the representative ILDs, which is considered a disease of aging.^[5]^ Interstitial lung abnormalities (ILAs) are radiological abnormalities found incidentally on chest computed tomography (CT) which are potentially related to ILD; they are considered as the early or mild form of IPF.^[6]^ ILAs occur in 2% to 7% of asymptomatic adults after imaging examination and it is important to identify the subpleural fibrotic subtypes, named as peripheral interstitial lung abnormalities (PILAs) (including interlobular septal thickening, ILST; intralobular interstitial thickening, ILIT; reticular shadow, RS; subpleural line, SL; and ground-glass opacity [GGO]).^[7,8]^ Winter et al analyzed the CT signs of 71 nonsmoking asymptomatic healthy volunteers (47 aged over 65 years and 24 young people aged 30–50 years). They found that the signs of GGO (25.5%), SL (21.3%), and RS (19.3%) were more prevalent among the elderly.^[9]^ Although it is usually asymptomatic, ILAs may be more likely to progress and to be associated with higher mortality in the elderly.^[10–12]^ The ILDs are related to the increased risk of lung cancer and complications related to lung cancer treatment, which can be predicted by CT-based radiomics signatures.^[13–15]^ Therefore, it is vital to distinguish “healthy aging” from diseases. The establishment of the “normal” lung appearance especially ILAs in the nonsmoking asymptomatic elderly cohort may avoid misdiagnosis and over-treatment of pulmonary diseases.

According to the report of World Health Statistics 2021, diabetes (2 million) and chronic respiratory diseases (4.1 million) are one of the top 10 leading causes of death in the world in 2019 and obesity and hypertension are 2 major health risk factors that need intervention. The global incidence of obesity increased to 13.1% in 2016, and the age-standardized prevalence of hypertension in adults was 22.1% worldwide in 2015, compared with 19.2% in China.^[16]^ A cohort study by Rossi et al compared the association of obesity and fat distributing with lung function in 957 men (73.6 ± 2.8 years) and 1024 women (73.2 ± 2.8 years).They conducted that weight gain with fat deposition accelerated age-related decline in lung function.^[17]^ Therefore, body mass index (BMI), blood glucose, blood pressure (BP), blood lipids and other indicators are selected as influencing factors for analysis.

The purpose of this retrospective study is to investigate subtle PILAs in nonsmoking asymptomatic urban cohort in China, analyze the imaging features and the related influencing factors of PILAs causing “normal aging” by low-dose computed tomography (LDCT), and in order to improve the understanding of peripheral interstitial lung aging.

## 2. Material and methods

### 2.1. Study participants

The subjects who underwent pulmonary LDCT in the Department of Radiology and Physical Examination Center of the First Hospital of Hebei Medical University (Shijiazhuang, China) from January 2017 to December 2019 were included in this retrospective study. As in the study of Li et al,^[18]^ the inclusion criteria were as follows: 18 to 80 years old; no smoking history; no chest signs or symptoms; and urban dwellers (lived in the city continuously for more than 15 years^[9]^ and no <6 months each year). The exclusion criteria^[9]^ were as follows: with respiratory symptoms such as cough, expectoration, or dyspnea; with pulmonary diseases, such as acute or chronic pneumonia, chronic obstructive pulmonary disease, pneumothorax, pleural effusion, atelectasis, thoracic deformity, asthma, pulmonary interstitial fibrosis, pulmonary tuberculosis, or lung tumor and radiotherapy; with abnormal pulmonary function; with history of chest trauma, operation, lung occupational, or other respiratory movement restriction diseases; with pulmonary heart disease or other organic heart disease; In the process of scanning, the breathing instructions could not be carried out correctly, and the image quality was poor. The Ethics Committee of the First Hospital of Hebei Medical University approved this study. This is a retrospective study in which the data are anonymous, and the requirement for informed consent was therefore waived.

### 2.2. CT examination

Neusoft 64-Slice spiral CT (NeuViz64 In, Neusoft, Shenyang, China) and Toshiba 320-Slice spiral CT (Aquilion ONE, Toshiba, Otawara, Japan) were used to acquire pulmonary images. The subjects were placed in the supine position with 2 arms raised above head. In order to exclude the interference of the volume efficiency of pulmonary blood, some subjects were placed in the prone position. Each volunteer was instructed to hold breath during full inspiration, and non-contrast LDCT scanning was undertaken from the thoracic inlet to the costophrenic angle. The scanning parameters of the Neusoft 64-Slice spiral CT were as follows: voltage, 120kV; current, 60mA; matrix, 512 × 512; the collimation 0.625 × 32 mm; reconstruction thickness, 1.25 mm; reconstruction interval, 1.0 mm; and the F50 reconstruction kernel. The scanning parameters of the other were as follows: voltage, 120kV; Sure EXP 3D, quality (Auto mAs); matrix, 512 × 512; collimation 0.5 × 40 mm; reconstruction thickness, 1.0 mm; reconstruction interval, 0.8 mm; and FC52 reconstruction kernel.

### 2.3. CT analysis

Five CT signs of PILA were selected: ILST, ILIT, GGO, RS, and SL. These CT signs of PILA were defined according to the nomenclature recommended by the Fleischner Society.^[19]^ The selected signs were quantified at 6 levels: thoracic inlet; aortic arch; above the tracheal carina; pulmonary hilum; right inferior pulmonary vein; 1 cm above the top of the right diaphragm. According to the distance to the pleura, the whole lung was divided into the central and subpleural areas, and the distribution of all 5 CT signs was analyzed. Three thoracic radiologists (with 10 and 15 years’ experience with thoracic CT, respectively) who were blinded to the clinical data independently scored all sections for the presence of disease. The presence of 5 CT signs was resolved by consensus. To ensure the consistency of the measurements, the data of 120 subjects (20 for each group) were premeasured, and the intraclass correlation coefficient was used for statistical analysis.

### 2.4. Clinical and blood biochemistry parameters

BP (systolic blood pressure ≧140 mm Hg and/or diastolic blood pressure ≧90 mm Hg would be diagnosed as hypertension^[20]^) and BMI (normal N, BMI < 25 kg/m^2^; overweight O, BMI ≧ 25–29.9 kg/m^2^; obese Ob, BMI ≧ 30 kg/m^2[21]^) were recorded and analyzed.

Blood biochemistry parameters were measured using the Beckman Coulter AU5821 automatic biochemistry analyzer (Beckman Coulter, CA).The subjects with fasting serum concentrations of glucose ≧6.1 mmol/L were considered as hyperglycemic,^[22]^ while the subjects with 1 or more of the following blood parameters were considered as hyperlipidemic, total cholesterol ≧240 mg/dL, triglycerides ≧200 mm/dL, lipoprotein cholesterol < 40 mm/dL, or low-density lipoprotein cholesterol ≧160 mm/dL.^[23,24]^

### 2.5. Statistical analysis

SPSS version 24.0 (SPSS, Inc., Chicago, IL) was used for the statistical analyses. Continuous variables were expressed as mean ± standard deviation. Non-normally distributed data was tested by Mann–Whitney U test or Kruskal–Wallis H test. Binary Logistic regression analysis (Forward method) was performed to identify the factors associated with all 5 CT signs of PILA. *P* values < .05 were considered statistically significant.

## 3. Results

According to inclusion criteria and exclusion criteria, a total of 733 eligible subjects were eventually included. The intraclass correlation coefficient values of 3 senior radiologists for ILST, ILIT, GGO, RS, and SL were 0.872, 0.877, 0.861, 0.760, and 0.750.

### 3.1. The clinical and laboratory characteristics of the study participants

The clinical and laboratory characteristics of 733 subjects were summarized in Table 1. The incidences of overweight or obesity in males appeared to be higher than the females in all 6 age groups. Both male and female showed increasing proportions of hypertension, hyperglycemia, and hyperlipidemia to different extents with age.

**Table 1:** The clinical and laboratory characteristics of the 733 subjects.

G	Sex	BMI			BP		GLU		BL	
		N	O	Ob	N	H	N	H	Non-H	H
G1	M (n = 42)	21	12	9	37	5	41	1	30	12
	F (n = 43)	37	4	2	40	3	43	0	37	6
G2	M (n = 77)	31	27	19	64	13	72	5	36	41
	F (n = 48)	40	8	0	48	0	48	0	38	10
G3	M (n = 81)	33	36	12	62	19	70	11	25	56
	F (n = 50)	36	11	3	46	4	50	0	27	23
G4	M (n = 78)	28	29	21	42	36	53	25	29	49
	F (n = 51)	33	14	4	40	11	46	5	14	37
G5	M (n = 74)	28	33	13	42	32	46	28	36	38
	F (n = 59)	29	20	10	44	15	51	8	20	39
G6	M (n = 72)	33	28	11	35	37	49	23	41	31
	F (n = 58)	24	25	9	30	28	32	26	13	45

G1: 18–29 yr; G2: 30–39 yr; G3: 40–49 yr; G4: 50–59 yr; G5: 60–69 yr; G6: 70–79 yr.

BL = blood lipid, BMI = body mass index, BP = blood pressure, F = female, G = group, GLU = blood glucose, H = higher, M = male, N = normal, Non-H = non-hyperglycemia, O = overweight, Ob = obesity.

### 3.2. Effects of age on PILA

**Figure 1.**
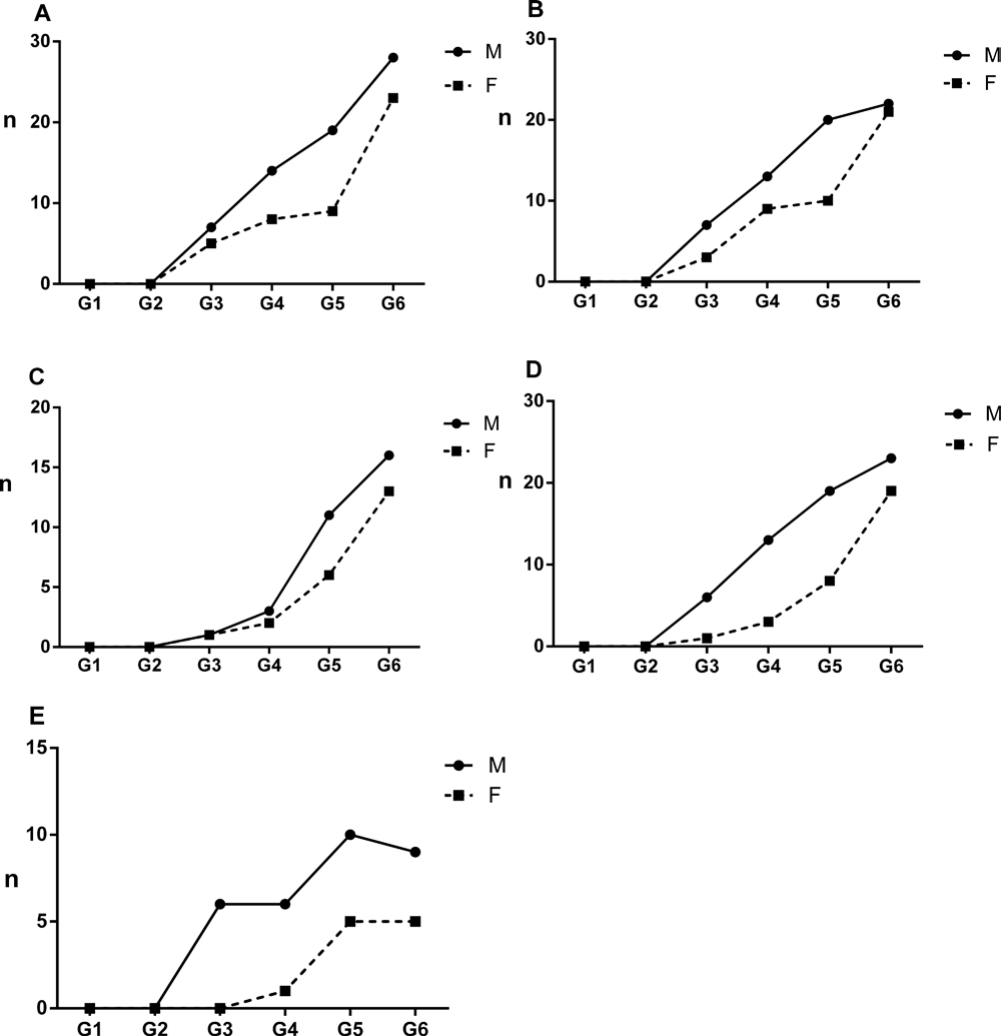
The distributions of ILST, ILIT, GGO, RS, and SL of males and females in different age groups. (A) ILST; (B) ILIT; (C) GGO; (D) RS; (E) SL. M: male; F: female; G1: 18 to 29 yr; G2: 30 to 39 yr; G3: 40 to 49 yr; G4: 50 to 59 yr; G5: 60 to 69 yr; G6: 70 to 79 yr. GGO = ground-glass opacity, ILIT = intralobular interstitial thickening, ILST = interlobular septal thickening, RS = reticular shadow, SL = subpleural line, TC = total cholesterol.

**Figure 2.**
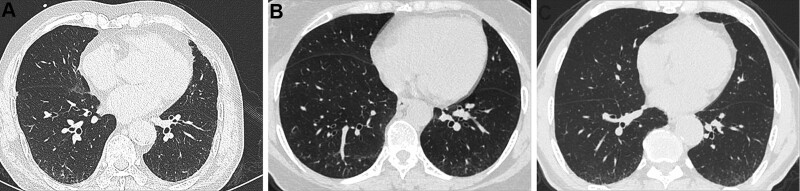
Representative CT images of ILST, ILIT, and RS of males and females in different age groups. (A) M, 57 yr, black arrow—ILST in subpleural area of lower lobe of right lung. (B) F, 61 yr, black arrow—ILST in subpleural area of lower lobe of left and right lung. (C) M, 78 yr, black arrow—ILST, ILIT and RS in subpleural area of lower lobe of left and right lung. CT = computed tomography, ILIT = intralobular interstitial thickening, ILST = interlobular septal thickening, RS = reticular shadow.

**Figure 3.**
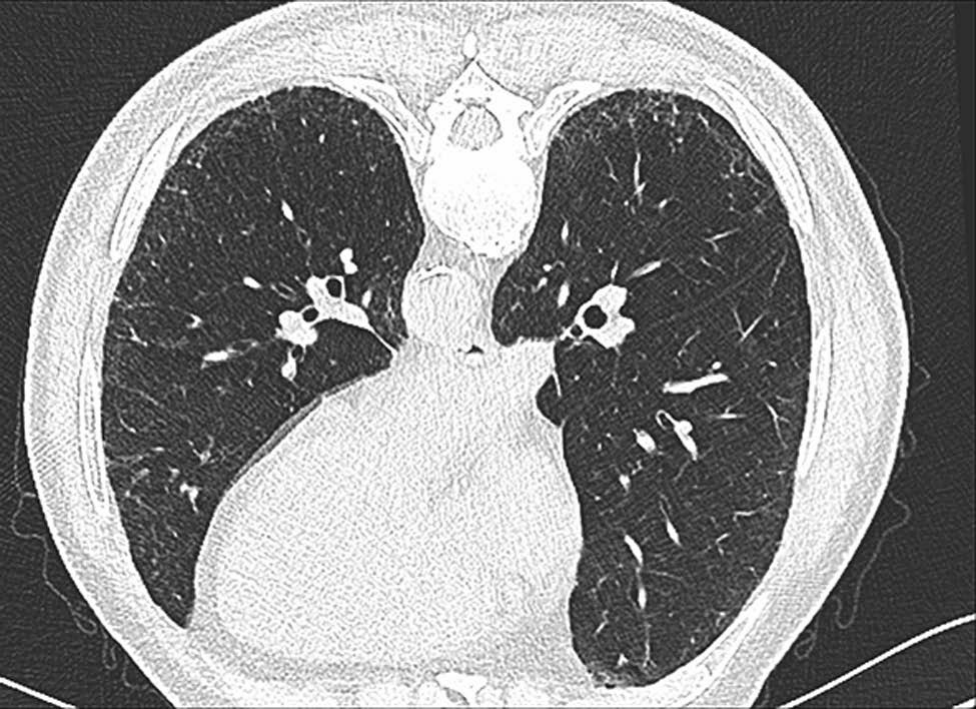
Representative CT image of GGO. M, 71yR, black arrow- GGO in subpleural area of lower lobe of left and right lung. CT = computed tomography, GGO = ground-glass opacity.

**Figure 4.**
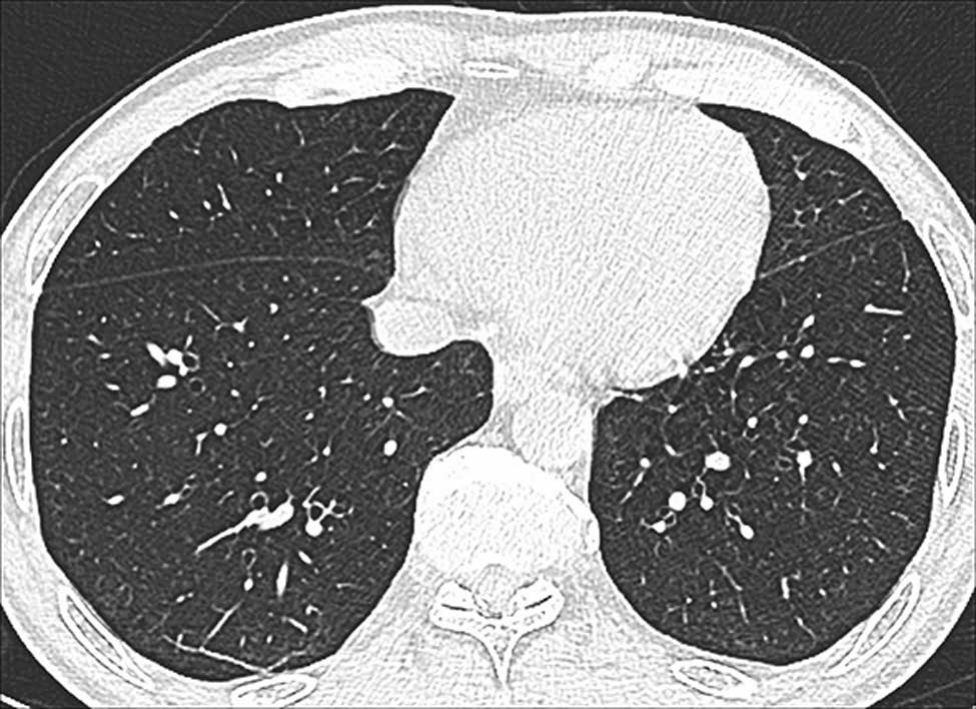
Representative CT images of SL. M, 53 yr, black arrow— subpleural line of lower lobe of right lung. CT = computed tomography, SL = subpleural line.

**Figure 5.**
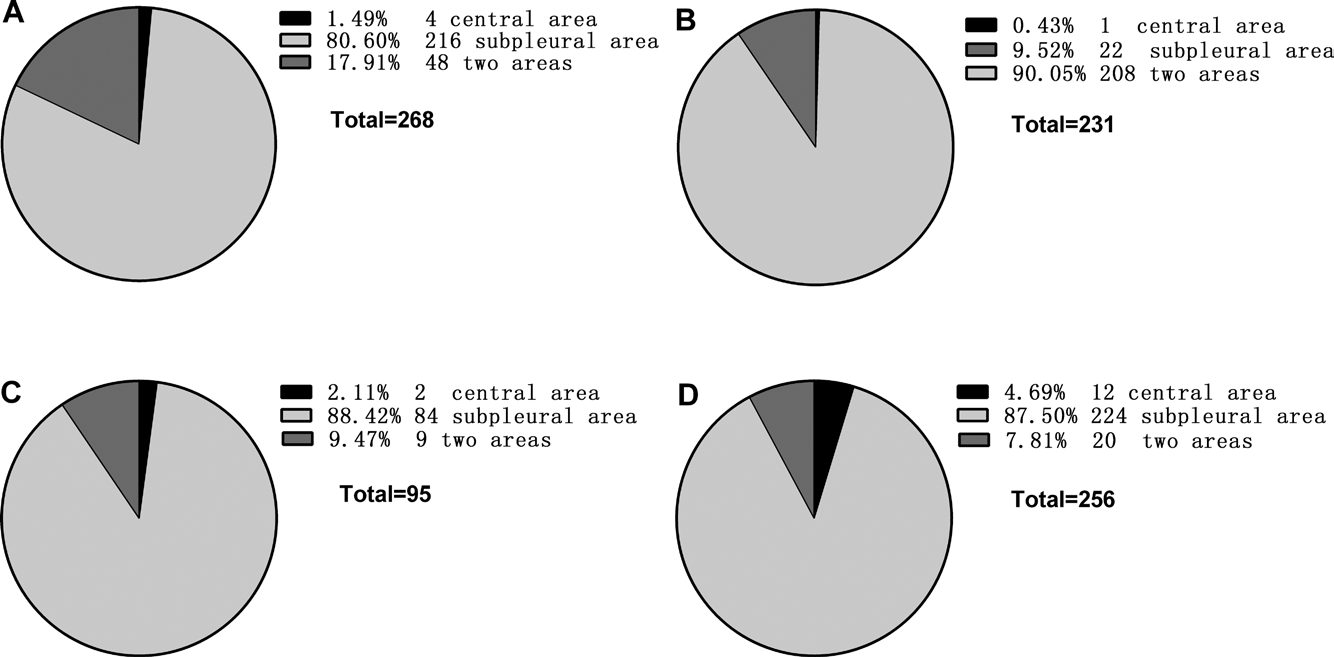
Central and peripheral distribution of ILST, ILIT, GGO, and RS at different levels. Central area: Outside 3 cm from pleura; Subpleural area: Inside 3 cm from pleura; 2 areas: Central and subpleural areas. Total: Total number of all levels. GGO = ground-glass opacity, ILIT = intralobular interstitial thickening, ILST = interlobular septal thickening, RS = reticular shadow.

**Figure 6.**
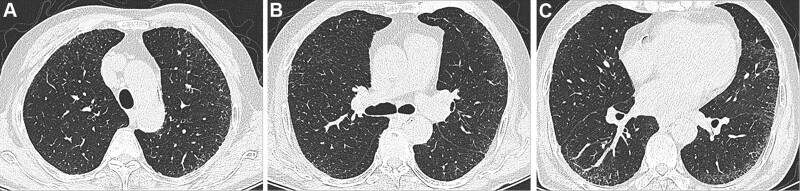
Representative CT images at different levels of the same volunteer. M, 70 yr—ILST, ILIT, and RS of bilateral subpleural area. (A) Upper lung level; (B) Middle lung level; (C) Lower lung level. CT = computed tomography, ILIT = intralobular interstitial thickening, ILST = interlobular septal thickening, RS = reticular shadow.

The CT signs of PILA in 733 nonsmoking asymptomatic urban residents were summarized in Table 2. None of the 5 CT signs was observed in the subjects below 40 years old, while the incidence of PILA increased with age in those over 40 years old (Fig. 1). The percentages of ILST (113/733, 15.4%, Fig. 2A), ILIT (105/733, 14.3%, Fig. 2B) and RS (92/733, 12.6%, Fig. 2C) were relatively higher, while the percentage of GGO (53/733, 7.23%, Fig. 3) and SL (42/733, 5.7%, Fig. 4) were lower. The 4 signs (GGO, ILST, ILIT, and RS) were mostly located in bilateral subpleural area of the lower lung (Figs. 5 and 6). Moreover, the CT signs in different age groups were significantly different (*P* all < .05). Specially, the proportions of all 5 CT signs in the elder (70–79 years) were up to 33.34% to 54.72%.

**Table 2:** The CT signs of PILA in 733 subjects.

G	Sex	GGO (n = 53)		ILST (n = 113)		ILIT (n = 105)		RS (n = 92)		SL (n = 42)	
		n	%	n	%	n	%	n	%	n	%
G1	M	0	0%	0	0%	0	0%	0	0%	0	0%
	F	0	0%	0	0%	0	0%	0	0%	0	0%
G2	M	0	0%	0	0%	0	0%	0	0%	0	0%
	F	0	0%	0	0%	0	0%	0	0%	0	0%
G3	M	1	1.89%	7	6.20%	7	6.67%	6	6.50%	6	14.29
	F	1	1.89%	5	4.42%	3	2.86%	1	1.09%	0	0%
G4	M	3	5.66%	14	12.39%	13	12.38%	13	14.13%	6	14.29%
	F	2	3.77%	8	7.08%	9	8.57%	3	3.26%	1	2.38%
G5	M	11	20.75%	19	16.81%	20	19.05%	19	20.65%	10	23.81%
	F	6	11.32%	9	7.97%	10	9.52%	8	8.70%	5	11.90%
G6	M	16	30.19%	28	24.78%	22	20.95%	23	25.00%	9	21.44%
	F	13	24.53%	23	20.35%	21	20.00%	19	20.65%	5	11.90%
*χ* ^2^		74.950		100.935		93.771		91.729		26.762	
*P*		.000*		.000*		.000*		.000*		.000*	

G1: 18–29 yr; G2: 30–39 yr; G3: 40–49 yr; G4: 50–59 yr; G5: 60–69 yr; G6: 70–79 yr.

CT = computed tomography, F = female, G = group, GGO = ground-glass opacity, ILIT = intralobular interstitial thickening, ILST = interlobular septal thickening, M = male, PILA = peripheral interstitial lung abnormalities, RS = reticular shadow, SL = subpleural line.

* *P < *.05.

The comparison of the 5 CT signs in different age groups was shown in Table 3. We found that there were no significant differences of all 5 CT signs among the 3 groups under 50 years old (18–29, 30–39, and 40–49 years), similar results between 50–59 and 60–69 age groups (*P* > .05). Accordingly, we compared the 3 groups aged 18 to 49, 50 to 69, and 70 to 79 (Table 3). The results demonstrated that all CT signs were significantly different among the subjects aged 18 to 49, 50 to 69, and 70 to 79 (*P* < .05).

**Table 3:** The comparison of CT signs of PILA indifferent age groups.

G	Statistic	GGO	ILST	ILIT	RS	SL
G1-G2	*Z*	0.000	0.000	0.000	0.000	0.000
	*P*	1.000	1.000	1.000	1.000	1.000
G1-G3	*Z*	-5.595	−41.9668	−27.977	−19.584	−16.786
	*P*	1.000	.343	1.000	1.000	1.000
G1-G4	*Z*	−14.205	−53.981	−65.345	−39.775	−19.888
	*P*	1.000	.053	.004*	.270	1.000
G1-G5	*Z*	−46.846	−77.158	−79.914	−74.402	−41.335
	*P*	.006*	.000*	.000*	.000*	.007*
G1-G6	*Z*	−81.758	−143.781	−121.227	−118.408	−39.469
	*P*	.000*	.000*	.000*	.000*	.014*
G2-G3	*Z*	−5.595	−41.966	−27.977	−19.584	−16.786
	*P*	1.000	.169	1.000	1.000	1.000
G2-G4	*Z*	−14.205	−53.981	−65.345	−39.775	−19.888
	*P*	1.000	.017*	.001*	.127	.945
G2-G5	*Z*	−46.846	−77.158	−79.914	−74.402	−41.335
	*P*	.001*	.000*	.000*	.000*	.001*
G2-G6	*Z*	−81.758	−143.781	−121.227	−118.408	−39.469
	*P*	.000*	.000*	.000*	.000*	.003*
G3-G4	*Z*	−8.610	−12.015	−37.368	−20.191	−3.101
	*P*	1.000	1.000	.286	1.000	1.000
G3-G5	*Z*	−41.250	−35.192	−51.936	−54.818	−24.548
	*P*	.006*	.463	.015*	.003*	.290
G3-G6	*Z*	−76.162	−91.815	−93.250	−98.824	−22.683
	*P*	.000*	.000*	.000*	.000*	.474
G4-G5	*Z*	−32.640	−23.177	−14.569	−34.627	−21.447
	*P*	.081	1.000	1.000	.299	.626
G4-G6	*Z*	−67.552	−89.800	−55.882	−78.632	−19.582
	*P*	.000*	.000*	.007*	.000*	.968
G5-G6	*Z*	−34.912	−66.623	−41.313	−44.005	1.865
	*P*	.043*	.001*	.137	.045*	1.000
G1-2-G3-6	*Z*	−6.713	−6.414	−7.319	−7.010	−4.227
	*P*	.000*	.000*	.000*	.000*	.000*
G1-3-G4-6	*Z*	−41.905	−72.699	−80.206	−81.607	−40.311
	*P*	.000*	.000*	.000*	.000*	.000*
G1-3-G4-5	*Z*	−23.611	−16.968	−31.939	−26.948	-
	*P*	.000*	.423	.013*	.032*	-
G1-3-G6	*Z*	−72.069	−72.646	−66.534	−70.511	-
	*P*	.000*	.000*	.000*	.000*	-
G4-5-G6	*Z*	−14.818	−55.678	−34.595	−43.563	-
	*P*	.000*	.000*	.006*	.000*	-

G1: 18–29 yr; G2: 30–39 yr; G3: 40–49 yr; G4: 50–59 yr; G5: 60–69 yr; G6: 70–79 yr; G1-3: 18–49 yr; G4-6: 50–79 yr; G4-5: 50–69 yr.

CT = computed tomography, G = group, GGO = ground-glass opacity, ILIT = intralobular interstitial thickening, ILST = interlobular septal thickening, RS = reticular shadow, PILA = peripheral interstitial lung abnormalities, SL = Subpleural line.

-: There was no statistical difference between 2 groups.

* *P < *.05.

### 3.3. Effects of sex on PILA

The CT signs of ILST, ILIT, GGO, RS, and SL were appeared slightly more in male subjects than those in female (Table 2 and Fig. 1), but there was no significant sex difference in GGO, ILST, ILIT and SL (*P* ≥ .05). The statistically difference only showed in RS aged 50 to 59 (*Z* = −2.615, *P* = .009)(Table 4).

**Table 4:** The comparison of CT signs of PILA between males and females.

G(M-F)	Statistics	GGO	ILST	ILIT	RS	SL
G1	*Z*	0.000	0.000	0.000	0.000	0.000
	*P*	1.000	1.000	1.000	1.000	1.000
G2	*Z*	0.000	0.000	0.000	0.000	0.000
	*P*	1.000	1.000	1.000	1.000	1.000
G3	*Z*	0.120	−1.237	−0.581	−1.332	−1.963
	*P*	.730	.216	.561	.182	.050
G4	*Z*	0.000	−1.271	−1.810	−2.615	−1.400
	*P*	.983	.204	.070	.009*	.162
G5	*Z*	0.644	−1.459	−1.627	−1.719	−0.909
	*P*	.422	.145	.104	.086	.363
G6	*Z*	0.001	−0.089	−0.678	−0.098	−0.707
	*P*	.979	.929	.498	.922	.480

G1: 18–29 yr; G2: 30–39 yr; G3: 40–49 yr; G4: 50–59 yr; G5: 60–69 yr; G6: 70–79 yr.

CT = computed tomography, F = female, G = group, GGO = ground-glass opacity, ILIT = intralobular interstitial thickening, ILST = interlobular septal thickening, M = male, PILA = peripheral interstitial lung abnormalities, RS = reticular shadow, SL = subpleural line.

* *P < *.05.

### 3.4. Analysis of related influencing factors on PILA

Binary Logistic regression analysis was used to predict ILST, ILIT, GGO, RS, and SL using the factors including age, sex, BMI, BP, and laboratory biochemistry parameters (GLU, total cholesterol, triglycerides, lipoprotein cholesterol, and low-density lipoprotein cholesterol). The results of ILST, ILIT and RS appeared goodness of fit (*χ*^2^ = 11.650, *P* = .167; *χ*^2^ = 8.945, *P* = .347; *χ*^2^ = 11.936, *P* = .154), while GGO and SL were on the contrary (*χ*^2^ = 19.178, *P* = .014; *χ*^2^ = 18.243, *P* = .018). Only age had a significant effect on ILST, ILIT and RS (Table 5).

**Table 5:** Binary logistic regression analysis of ILST, ILIT, and RS.

Variable	B	Standard error	Wald	*P*	OR	95%CI (LL-UL)	
ILST							
Constant	−5.919	19.338	0.094	.760	0.003		
Age*	0.081	0.010	65.252	.000	1.085	1.063	1.106
ILIT							
Constant	−12.110	19.403	0.390	.533	0.000		
Age*	0.069	0.010	48.627	.000	1.071	1.051	1.092
RS							
Constant	−9.674	21.316	0.206	.650	0.000		
Age*	0.092	0.012	57.410	.000	1.097	1.071	1.123

Only the influencing factors related to CT signs were listed in the table, and the non-influencing factors were omitted, such as Sex, BMI, BP, GLU, TC, TG, HDL-C, and LDL-C.

BMI = body mass index, BP = blood pressure, GLU = blood glucose, HDL-C = high density lipoprotein cholesterol, ILIT = intralobular interstitial thickening, ILST = interlobular septal thickening, LDL-C = low density lipoprotein cholesterol, RS = reticular shadow, TC = total cholesterol, TG = triglyceride.

* *P < *.05.

## 4. Discussion

This study aimed to investigate the CT characteristics of peripheral interstitial lung aging induced by “normal aging” in a nonsmoking asymptomatic urban cohort in China and the related influencing factors (age, sex, BMI, hypertension, hyperglycemia, and hyperlipidemic) analyses. We found that there were significant differences in PILA among different age groups, which meant age had an effect on PILA. Among the participants under 40 years old, no signs (GGO, ILST, ILIT, RS, and SL) were observed, while in those over 40 years old, the incidence of PILA increased with age. Interestingly, all these CT signs showed significant differences among the groups aged 18 to 49, 50 to 69, and 70 to 79. Thus, we speculated that the age of 40 may be the threshold of the development of PILA, and the age of 50 may be the reference age of peripheral interstitial lung aging. However, there was no significant sex difference in PILA, and hypertension, hyperglycemia and hyperlipidemia had no obvious effects on peripheral interstitial lung aging.

There is increasing awareness of the clinical significance of ILAs incidentally detected on chest CT.^[25]^ A recent study has confirmed that the ILAs is not a benign finding. Studies by Araki et al demonstrated that ILAs can progress over time. In this study, up to 3 quarters of subjects with ILAs progressed, and that progression in subjects with ILAs was associated with a faster decline in pulmonary function and a higher risk of death.^[26]^ A possible relationship between ILAs and pulmonary fibrosis has been confirmed. In histological evaluation, it was found that after routine follow-up, the incidence of patients with ILAs progressing to pulmonary fibrosis increased overall.^[27]^ As the development of anti-fibrosis treatment for patients with IPF, it is particularly important to identify patients with ILAs for effective management.^[28]^ Age is an important factor affecting PILA. The reason may be related to the anatomy and pathophysiology of pulmonary peripheral interstitial. Some studies in asymptomatic “healthy” people have confirmed the age-related structural changes of the respiratory system.^[29]^ First, the age-related changes in thoracic anatomy can affect pulmonary interstitial aging. Kyphosis: Woods et al conducted that age played a significant role in kyphosis progression.^[30]^ The age-related changes in anatomical structure and intrinsic function of respiratory muscle: the age-related atrophy of muscle fibers, known as age-related degeneration of skeletal muscle, sarcopenia, includes loss of muscle content, mass, and strength.^[31]^ Wilkinson deemed that the loss of muscle mass occurred incipiently from middle age (1%/year), and could reach a loss of 50% by the 8 to 9th decade of life in severe instances.^[32]^ Porter et al studied the mitochondrial respiratory capacity and coupling control in skeletal muscle biopsies for young (28 ± 7 years) and older (62 ± 8 years) adults. They found that the mitochondrial respiratory capacity and coupling control of the skeletal muscle declined with age. Lower respiratory capacity and coupling efficiency resulted in a reduced capacity for ATP production in the skeletal muscle of older adults.^[33]^ Secondly, the age-related changes of lung structure can also affect pulmonary interstitial aging. Animal experiments and human studies have shown that collagen in lung tissue increases and elastic decreases with age, which leads to the increase of lung stiffness, the decrease of elasticity and compliance.^[34–36]^ Sicard et al studied the stiffness of lung tissue in 13 patients (11–60 years), and they found that the stiffness of both lung parenchyma and pulmonary vessel increased significantly with age, especially the pulmonary vessel.^[36]^ Cho et al deemed that the age-related decrease of elasticity was not uniform throughout the lung, and the compliance of each region of the lung was different, so the ventilation distribution may be uneven, which was one of the pathological foundations of GGO.^[37]^ Thirdly, the age-related decline in lung function and increase in functional residual capacity are also the causes of interstitial lung aging. A systematic review of prospective cohort studies by Thomas et al showed that lung function would decline from the age of 30 to 40 of healthy people, and the decline rate of FEV1 was about 17.7 to 46.4 mL/year, while that of FVC was about 14.11 to 65.66 mL/year.^[38]^ In our study, the incidence of all 5 CT signs of PILA increased with age. The incidence in the 70 to 79 years group was nearly 40% to 45%, and the PILAs were mainly located in the subpleural area of the lower lung. Copley et al compared the CT signs of the elder (40 cases, over 75 years old) and younger (16 cases, under 55 years old).They found that the occurrence percentage of RS and SL located in the subpleural area in the elderly group was 60% (24/40), while these signs were not found in the younger group.^[39]^ A 6-year follow-up longitudinal study of 66 volunteers (40 smokers and 26 nonsmokers) by Vikgren et al confirmed that the incidence of ILST in nonsmokers increased with age.^[40]^ Winter et al analyzed CT signs of 71 asymptomatic healthy volunteers (47 aged over 65 and 24 aged 30–50), and they found the incidences of RS and SL (19.1% and 21.3%, respectively) in the elderly group were significantly higher than those in the young group (0%, *P* < .05).^[9]^ These results support part of our study.

In our study, the incidences of ILST, ILIT, GGO, RS, and SL in CT were slightly higher in male subjects than that in female subjects. There were statistically significant differences of sex in RS in 50 to 59 years groups. However, there was no significant sex difference in GGO, ILST, ILIT, and SL. In this case, compared with age, sex has less influence on interstitial lung aging. The differences can be explained by the aspects of anatomy and pathophysiology such as kyphosis and respiratory muscle changes. Lorbergs et al conducted a longitudinal study on the relationship between kyphosis and pulmonary function in 275 patients (82 males and 193 females) with an average age of 63 years. They found that with the increase of the severity of kyphosis, the FEV1 of patients decreased, especially in female.^[41]^ The kyphosis angles increased faster in females than males over 40 years old, an average of 43° aged 55 to 60 and 52° aged 76 to 80.^[42]^ According to the study of Mitchell et al, the muscle mass loss rate of females and males over 75 years old was different (0.64%–0.7% and 0.8%–0.98% per year, respectively), while the muscle strength loss rate was even faster (3%–4% per year in males and 2.5%–3% in females).^[43]^

There are several limitations in our study. First, this is a single-center, single-race study and the sample size is not very large. Second, there may be selection bias in this study toward selecting nonsmoking individuals without respiratory symptoms who may not represent a truly healthy population. Third, CT signs maybe a miscarriage.

## 5. Conclusion

LDCT can be used as a noninvasive method to evaluate the peripheral interstitial lung aging. PILA were mainly affected by age, while sex, BMI, BP and laboratory biochemistry parameters had little effect on PILA. PILA observed before the age of 40 years should be considered as an abnormal finding, whereas it is common in individuals over 70.

## Acknowledgments

Throughout the writing of this dissertation we have received a great deal of support and assistance. We gratefully acknowledge the assistance of Meibao Kong and Shengnan Li for collecting the clinical data of the volunteers.

## Author contributions

**Conceptualization:** Shujing Li, Yingqi Zhang.

**Data curation:** Zhimei Gao, Xin Li.

**Formal analysis:** Zhimei Gao, Chenguang Zhang.

**Funding acquisition:** Shujing Li, Yingqi Zhang.

**Investigation:** Yan Li, Chenguang Zhang.

**Methodology:** Yaguang Li.

**Project administration:** Yaguang Li, Mengyue Sun.

**Supervision:** Mengyue Sun, Yalan Wu.

**Validation:** Yalan Wu.

**Visualization:** Yan Li.

**Writing – original draft:** Zhimei Gao, Xin Li.

**Writing – review & editing:** Shujing Li.
